# Trimetallic CuO/Ag/NiO supported with silica nanoparticles based composite materials for green hydrogen production

**DOI:** 10.1038/s41598-023-43697-4

**Published:** 2023-10-07

**Authors:** Gowhar A. Naikoo, Mustri Bano, Israr U. Hassan, Mohd Monis Ayyub, Mona Zamani Pedram

**Affiliations:** 1 Department of Mathematics & Sciences, College of Arts & Applied Sciences, Dhofar University, PC 211 Salalah, Oman; 2 New Chemistry Unit and School of Advanced Materials, Jawaharlal Nehru Centre for Advanced Scientific Research, Bangalore, 560064 India; 3 Faculty of Mechanical Engineering-Energy Division, K.N. Toosi University of Technology, No. 15-19, Pardis St., Mollasadra Ave., Vanak Sq., P.O. Box: 19395-1999, Tehran, 1999 143344 Iran

**Keywords:** Energy science and technology, Materials science, Nanoscience and technology

## Abstract

Production and utilization of grey and blue hydrogen is responsible for emission of millions of tons of carbon dioxide (CO_2_) across the globe. This increased emission of CO_2_ has severe repercussions on the planet earth and in particular on climate change. Here in, we explored advance bimetallic (BM) CuO/Ag and trimetallic (TM) CuO/Ag/NiO based nanoporous materials supported with silica nanoparticles (SiNPs) via sol–gel route. The explored nanocatalysts were characterized by Powder X-ray diffraction (P-XRD), scanning electron microscopy (SEM), transmittance electron microscopy (TEM), X-ray photoelectron spectroscopy (XPS), energy dispersive X-ray spectroscopy (EDX), and Raman spectroscopic techniques. These advance nanocatalysts were evaluated for the green hydrogen production through electrocatalysis and photocatalysis. The catalysts exhibited an exceptional catalytic performance, the onset potential for hydrogen evolution reaction (HER) was determined to be − 0.9 V BMSiNPs-GCE and − 0.7 V (vs Ag/AgCl) for TMSiNPs-GCE, whereas η@10 for BMSiNPs-GCE and TMSiNPs-GCE is − 1.26 and − 1.00 V respectively. Significantly, the TMSiNPs composite and the BMSiNPs composite exhibited superior photochemical H2 evolution rates of 1970.72 mmol h^−1^ g^−1^ and 1513.97 mmol h^−1^ g^−1^, respectively. The TMSiNPs catalyst presents a highly promising material for HER. This study reveals a cost-effective approach to develop sustainable and resourceful electrocatalysts for HER.

## Introduction

Over the last century, there has been a substantial acceleration in the worldwide energy consumption attributed to the growth of the population and advancements in global development. Projections indicate a persistent trend of rising energy demand, with an anticipated increase from 16 Terawatts (TW) in 2010 to around 23 TW by 2030. By 2050, it may reach up to 30 TW^1^. The REN21 Global Status Report (GSR) 2022 shows that while renewable energy deployment is increasing, the shift to a fully sustainable energy system remains slow. Renewables made up 29.3% of global electricity and 12.4% of total energy in 2020, but conventional sources remain dominant at 79.5%. The transition to renewable energy must be accelerated for meeting climate goals and providing universal access to energy^2^. The heavy reliance on fossil fuels has not only resulted in their rapid depletion but has also caused significant environmental problems and contributed to global warming.

Molecular hydrogen gas (H2) is a desirable fuel alternative because of its high energy density per unit mass besides the fact that its only byproduct after combustion is non-contaminated water^3^. It is an outstanding energy carrier and has potential zero emission CO_2_ release in future and it is sustainable source of energy. However, it is unfortunate that the majority of H_2_ is now formed via steam reforming of fossil resources, a process that exhibits a measly conversion rate and releases CO_2_ into the atmosphere. This undoubtedly exacerbates the existing environmental issues. Consequently, it becomes imperative to urgently develop a method for H_2_ generation that is both clean and renewable, without contributing to further damage to our environment. Such a development is crucial for the positive establishment of a hydrogen economy^4^. On a more positive note, electrochemical water splitting, or water electrolysis, has gained recognition as a promising substitute to fossil fuels. This method is widely acknowledged for its environmental friendliness, efficiency, and sustainability. By employing water as both the initial molecule and the resulting byproduct in the hydrogen economy cycle, energy can be harnessed through the combustion of H_2_ while water is simultaneously regenerated. Water electrolysis, a process with its initial documentation dating back to 1789, has gained recognition as a promising alternative to fossil fuels^5^. It contains two separate half-cell reactions: the HER and the oxygen evolution reaction (OER). These reactions play a pivotal role in facilitating the electrolysis of water [H_2_O (l) → H2 (g) + 1/2O_2_ (g), ΔG° =  + 237.2 kJ mol^−1^, ΔE° = 1.23 V vs normal hydrogen electrode (NHE)]. This revolutionary process challenges the traditional methods of H2 production and presents a viable pathway towards a greener future.

Groundbreaking research in electrocatalytic HER via nanoparticles with metal oxides have shown promising results^4^. Nanoparticles have unique properties that make them ideal candidates for electrocatalytic applications, and their size and shape can be controlled to enhance their catalytic activity. Various metals, including platinum, nickel, cobalt, copper, and molybdenum, have been explored as catalysts, and their performance has been improved through surface modification and hybridization with other materials^6–10^. Platinum is the state of the art of this catalyst for hydrogen evolution due to its high durability and low onset potential, but its scarcity and limiting widespread application due to high costs. Therefore, first row d-block metals, such as copper, have been explored as an alternative^9^.

Copper, known for its abundance and cost-effectiveness, has emerged as a compelling option for electrocatalysts in the HER. Various research studies have supported the exceptional catalytic activity of copper-based composites in both electrocatalytic and photocatalytic processes for HER^11–15^. The development of efficient copper-based catalysts holds great significance in advancing large-scale hydrogen production. Recent efforts have focused on synthesizing and characterizing diverse copper-based materials, including bimetallic and trimetallic composites, for their application in HER^16–19^. Copper-based composites demonstrate exceptional electrocatalytic performance with high current densities and low overpotentials, positioning them as a viable substitute to platinum-based catalysts^8^. Additionally, the utilization of copper-based composites for HER offers an environmentally friendly solution to the pressing carbon emission challenge associated with hydrogen production. Unlike grey and blue hydrogen processes that contribute significantly to climate change through carbon dioxide emissions, copper-based composites provide a greener pathway with significantly reduced carbon emissions^20^. Several studies have been published that have investigated the field of hydrogen evolution reaction (HER) for hydrogen production, spanning a wide variety of materials with varied dimensionalities, such as zero-dimensional (0D), one-dimensional (1D), two-dimensional (2D), and three-dimensional (3D) structures. These investigations have delved into topics such as the design of materials, synthesis methodologies, characterization techniques, catalytic mechanism investigation, and performance validation or optimization^21–23^.

In this study, advanced BM-CuO/Ag and TM-CuO/Ag/NiO-based nanoporous materials supported with silica nanoparticles (SiNPs) were explored via sol–gel route for green hydrogen production by splitting water. The characterization techniques confirmed the successful synthesis of (bimetallic CuO/Ag/SiNPs) BMSiNPs and (trimetallic CuO/Ag/NiO/SiNPs) TMSiNPs nanoporous composite materials. The study revealed significant findings regarding the HER in various composite materials. The onset potential for HER was determined to be ‒ 0.9 V for BMSiNPs-GCE and ‒ 0.7 V (vs Ag/AgCl) for TMSiNPs-GCE. The overpotential at a current density of 10 mA cm^−2^ was found to be ‒ 1.26 V for BMSiNPs-GCE and ‒ 1.00 V for TMSiNPs-GCE. Tafel slopes were calculated as 294 mV dec^−1^ for BMSiNPs-GCE and 270 mV dec mV dec^−1^ for TMSiNPs-GCE. Notably, the composite materials, TMSiNPs and BMSiNPs, demonstrated superior rates of photochemical H_2_ evolution. The TMSiNPs composite exhibited a remarkable rate of 1970.72 mmol h^−1^ g^−1^, while the BMSiNPs composite displayed a slightly lower but still impressive rate of 1513.97 mmol h^−1^ g^−1^. These findings highlight the commendable performance of these composites in efficiently generating hydrogen gas through photochemical processes. The TMSiNPs catalyst demonstrates promising potential for the HER. Compared to the bimetallic counterpart, the trimetallic composite exhibits superior electrochemical and photochemical HER activity. This enhanced performance is attributed to efficient charge carrier separation and increased active surface area. These findings emphasize the effectiveness of trimetallic composites as catalysts for HER. Further research is necessary to gain deeper insights into the involved mechanisms and explore their applications in sustainable hydrogen production.

## Methods

### Synthesis of nanoporous BMSiNPs and TMSiNPs composite materials

In the synthesis process of BMSiNPs, a mixture of 2 g of Cu(NO_3_)_2_ and 2 g of ultrapure water (57.12 wt%, dispersed in 42.88% water) was prepared. Subsequently, 4 g of Brij S 20 was incorporated into the solution. Following thorough mixing, the solution was left to react for a duration of 5 mins. In a separate step, 2 g of AgNO_3_ (57.12 wt%, Sigma Aldrich) was dissolved in 2.0 g of ultrapure water and gradually added to the previously mentioned solution. Subsequently, 0.5 mL of SiNPs (Sigma Aldrich, LUDOX HS-40) was incorporated into the mixture, which was then subjected to stirring for a period of 30 mins, resulting in the formation of a dark blue paste. Lastly, the paste underwent aging and calcination at a temperature of 500 °C, utilizing a heating system and cooling system rate of 4.17 °C min^−1^.

**Figure 1:**
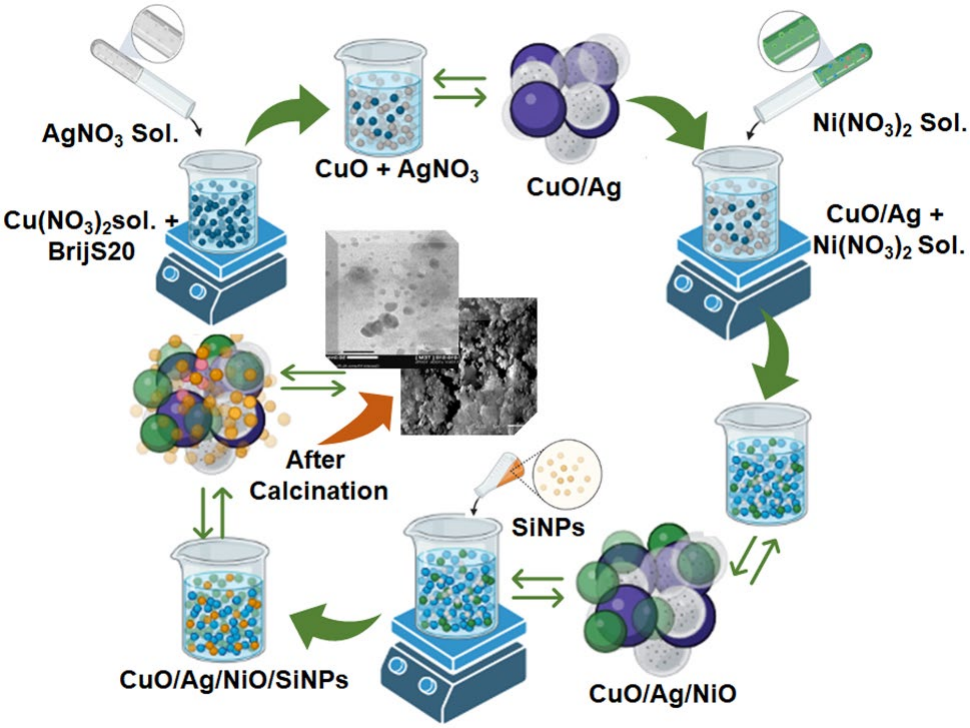
Synthesis representation of TMSiNPs.

The TMSiNPs gel was synthesized by mixing 2 g of Cu(NO_3_)_2_ (57.12 wt%, suspended in 42.88% water), 4 g of Brij S 20 (15.00 wt%, dispersed in 85.00 wt% water), 2 g of AgNO_3_ (57.12 wt%, dispersed in 42.86% water), 2 g of Ni(NO_3_)_2_ (57.12 wt%), and 1.5 g of ultrapure water (42.85 wt%). The above-mentioned materials were mixed according to the stepwise protocol described earlier. Additionally, 0.5 mL of SiNPs was mixed with the mixture. The resulting mixture was subjected to aging and calcination following the previously described procedure. It is worth noting that the surfactant Brij S 20, which comprises a hydrophilic polyethylene glycol head and a hydrophobic hydrocarbon tail, plays a crucial role in the synthesis of nanoporous materials. These surfactant molecules adsorb onto the surface of the materials, preventing aggregation and the formation of nanoparticles (Fig. 1)^24^.

### Materials characterization

The P-XRD analysis employed Cu Ka radiation and was conducted using the X Pert PRO X-ray Diffraction instrument. The SEM and EDX studies were investigated using a JEOL JSM-6510LA electron microscope, while a JEOL Model JEM1400 instrument was used for the TEM study. The materials were synthesized using a HD150 PAD model Muffle Furnace, and the Autolab PGSTAT204 FRA32M was utilized to carry out CV and DPV studies. The Raman spectra were obtained from various locations of the sample using a Jobin Yvon LabRam HR spectrometer equipped with Ar laser 632 nm. XPS measurements were conducted via Omicron spectrometer with Al Ka as the X-ray source (1486.6 eV) to examine the atomic ratio of the elements and determine the corresponding elemental composition of the samples.

### Preparation of modified electrode for HER

In the experimental configuration, room temperature electrochemical measurements were conducted using a set of three electrodes: an Ag/AgCl reference electrode (RE), a glassy carbon working electrode (GCE) with a diameter of 5.0 mm and a Pt counter electrode (CE). To modify the GCE surface, the initial glassy carbon electrode (b-GCE) was subjected to treatment with a dispersion consisting of BMSiNPs-GCE and TMSiNPs-GCE. The dispersion was prepared by subjecting 5 mg of each nanoporous materials to sonication in 10 mL of ethanol. Before the alteration of the electrode, meticulous steps were taken, including polishing the GCE surface with alumina slurry, subjecting it to ethanol sonication for 10 mins, rinsing it with distilled water, allowing it to dry at room temperature, and performing a final cleaning.

The dispersed mixture was then drop-casted onto the GCE surface, allowing the solvent to evaporate naturally at ambient temperature. To optimize the electrode performance, the modified GCEs underwent electrochemical activation through cyclic potential sweeps in − 1.0 to + 2.0 V range in a 0.1 M HNO_3_ solution. This process aimed to attain a stable voltammogram. After each electrochemical study, the electrode surface was purified by carrying out cyclic sweeps in the reverse direction (from 1.0 to 0.0 V) in a 1 mM NaOH solution. The obtained voltammograms were subsequently calibrated to the potential versus reversible hydrogen electrode (RHE) via a specific equation.

1 $${\text{E vs}}.{\text{ RHE }} = {\text{ E vs }}\left( {{\text{Ag}}/{\text{AgCl}}} \right) \, + \, 0.0{\text{59 pH }} + \, 0.{199 }\left( {\text{V}} \right)$$

## Results and discussion

**Figure 2:**
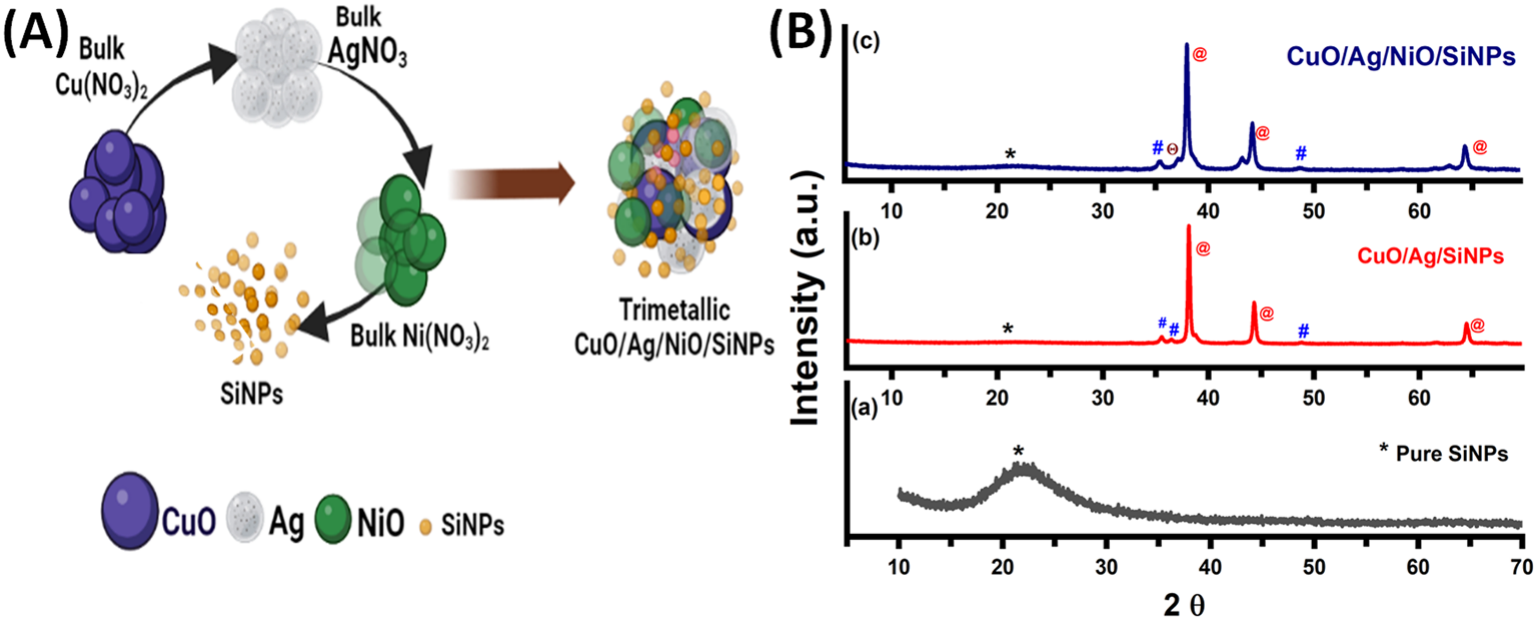
(**A**) Schematic representation of TMSiNPs formation, (**B**) XRD Results of (a) Pure SiNPs (b) BMSiNPs (c) TMSiNPs.

The synthesis of TMSiNPs composite shows in Fig. 2A, a sequence of intricate chemical process ensues, foremost to the formation of a highly stable and efficient catalytic material. The SiNPs, being highly porous, deliver a large surface area aimed at the interaction among the metal oxides and the surrounding environment. The CuO, Ag, and NiO, being highly oxidative and reducible in nature, readily react with each other to form a composite material. P−XRD analysis of the synthesized BMSiNPs and TMSiNPs composite material showed the presence of distinct diffraction peaks, indicating the successful formation of the composite (Fig. 2B). For BMSiNPs, strong reflections are observed from the (111), (200) and (220) planes of the face-centered cubic (FCC) structure of CuO (JCPDS file no. 03-065-3288) at 36°, 50° and 62°, respectively. Additionally, intense peaks at 38°, 45°, 64°, 77°, 82° correspond to the (111), (200), (220), (311) and (222) planes of Ag FCC structure (JCPDS no. 4.783) and a slight SiNPs hump at 22° (Fig. 2B(b))^24^.

For TMSiNPs, the XRD pattern exhibited several prominent peaks at 2θ values of 38.2°, 44.6°, and 64.5°, which can be attributed to the (111), (200), and (220) crystal planes of face-centered cubic (fcc) silver (Ag), respectively. Additionally, diffraction peaks at 2θ values of 36.3°, and 48.0° correspond to the (111), and (200) planes of CuO, respectively. Furthermore, the diffraction pattern also showed the presence of peaks at 2θ values of 48.3°, 43.2°, and 64.1°, which can be attributed to the (111), (200), and (220) planes of nickel oxide (NiO) with a slight SiNPs hump at 22°, respectively (Fig. 2B(c)). These findings suggest the presence of SiNPs and Ag distributed in the CuO and NiO matrix. Notably, the absence of any other peaks implies a high degree of crystallinity and the absence of noteworthy flaws or contaminants in the composite.

**Figure 3:**
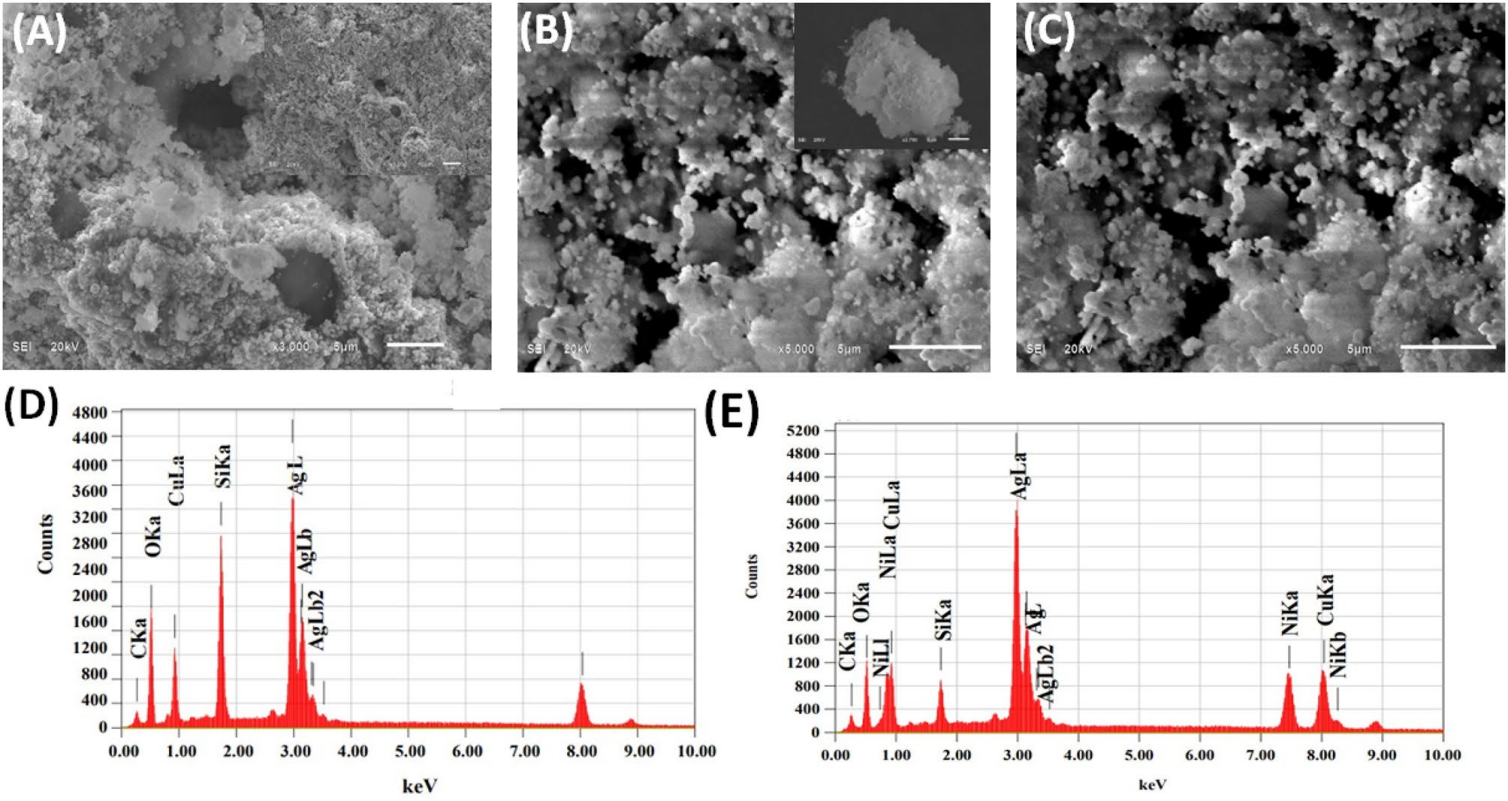
SEM images of (**A**) BMSiNPs at 5 µm, (**B**, **C**) TMSiNPs at 5 µm. EDX spectra of (**D**) BMSiNPs and (**E**) TMSiNPs.

The study involves the characterization of nanoporous material made of TMSiNPs using SEM and TEM imaging. In the case of BMSiNPs with surfactant Brij S 20, it has been observed that even after the calcination process, the surfactant molecules remain adsorbed onto the surface of the nanoparticles. This results in the formation of minimal nanoparticle formation, as shown in Fig. 3A at 5 µm. On the other hand, in the case of TMSiNPs with surfactant Brij S 20 (Fig. 3B,C), the inclusion of NiO increases the porosity of the material and surfactant Brij S 20 acts as a pore-forming agent throughout the calcination process, and the presence of NiO further enhances the pore-forming ability of the surfactant. The NiO nanoparticles act as templates for the formation of pores in the final product, resulting in a higher degree of porosity. With the increase in temperature, the surfactant molecules start to decompose and generate carbonaceous residues. These residues eventually burn off at high temperatures, leaving behind pores in the nanoparticles. Figure 3D and E EDX analysis supports the presence of C in the nanoparticle assembly, indicating the presence of the surfactant fragments in the nanoparticle structure, which supports the generation of porosity by the decomposition of the surfactant Brij S 20. Figure 3D and E illustrates the distribution of the elemental composition for BMSiNPs and TMSiNPs. The peaks for Cu, Si, Ag, Ni, C and O were observed with high intensity of TMSiNPs. No other elemental impurities were detected.

The TEM study of BMSiNPs at 500 nm show a bulk nanoporous image that is consistent with the SEM image because the particles in this case are relatively large, and they are not expected to exhibit significant variation at the nanoscale Fig. S1A. On the other hand, in the case of TMSiNPs, Fig. S1B and C shows the presence of nanoparticles is evident in the TEM images at 100 nm and 50 nm due to the reduced size of the particles. The histogram result for TEM images Fig. S1B and C of TMSiNPs show the frequency distribution of particle sizes, with the peak of the distribution indicating the most common particle size in the sample. In this case, the histogram analysis of TEM images Fig. S1B and C show that the usual particle size of TMSiNPs is around 12 nm and 19 nm, respectively. The presence of NiO in the nanoparticle structure is expected to influence the particle size, as NiO can act as a nucleating agent for nanoparticle formation.

TMSiNPs have a complex chemical environment that can be analysed by XPS. The wide spectrum XPS of TMSiNPs shows the existence of Ag, Cu, Ni, Si, and O elements in the nanoparticles. A combined graph of the XPS spectra of TMSiNPs reveals the binding energy of each element in the nanoparticles, which is useful in understanding the surface chemistry of the nanocomposite. The Cu 2*p* spectrum shows two foremost peaks at 932.8 eV and 952.6 eV, which resemble to Cu 2*p*3/2 and Cu 2*p*1/2 binding energies, respectively which indicate the presence of Cu in the + 2 oxidation state. The Ag 3*d* spectrum shows two main peaks at 368.2 eV and 374.2 eV, which correspond to Ag 3*d*5/2 and Ag 3*d*3/2 binding energies, respectively, indicating the presence of Ag in the metallic state (0 oxidation state). The Ni 2*p* spectrum shows two main peaks at 853.5 eV and 871.1 eV, which correspond to Ni 2*p*3/2 and Ni 2*p*1/2 binding energies, respectively, indicating the presence of Ni in the + 2 oxidation state. The Si–O 2*p* spectrum shows a main peak at 103.6 eV, which corresponds to the Si–O binding energy in SiNPs, indicating the presence of Si in the + 4 oxidation state. The O 1*s* spectrum shows a main peak at 530.1 eV, which indicating the presence of O in the − 2 oxidation state (Fig. S2A–F)^24,25^.

**Figure 4:**
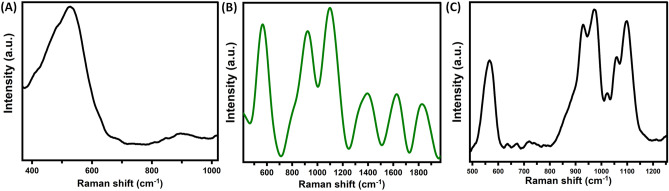
Raman spectra of (**A**) pure SiNPs, (**B**) BMSiNPs and (**C**) TMSiNPs.

Raman spectra of various samples including Fig. 4A pure SiNPs, (B) BMSiNPs, and (C) TMSiNPs, exhibit characteristic peaks that correspond to the vibrational modes of the materials present. The Raman spectra of pure SiNPs show a distinctive peak around 520 cm^−1^, which corresponds to the Si–O–Si vibrational mode. The peaks at 502 cm^−1^ at corresponds to the symmetric stretching vibration of the Si–O–Si bond, 922 cm^−1^ corresponds to the symmetric stretching vibration of the Cu–O bond, and the peak at 1095 cm^−1^ corresponds to the asymmetric stretching vibration of the same bond, suggesting the presence of CuO in the sample. The peak at 1395 cm^−1^ corresponds to the asymmetric stretching vibration of the Cu–O bond, providing additional evidence of the presence of CuO. The peak at 1403 cm^−1^ corresponds to the stretching vibration of the Ag–O bond, demonstrating the presence of Ag in the sample. Finally, the peaks at 1637 cm^−1^ and 1828 cm^−1^ correspond to the stretching vibrations of the C=C bond and the C=O bond, respectively, which are characteristic peaks of BMSiNPs (Fig. 4B). Finally, the Raman spectra of TMSiNPs (Fig. 4C) exhibit distinctive peaks corresponding to CuO, Ag, NiO, and SiNPs. The intense peak at 567 cm^−1^ corresponds to the symmetric stretching vibration of the Si–O bond, the peak at 929 cm^−1^ corresponds to the symmetric stretching vibration of the Ni–O bond, while the peak at 976 cm^−1^ corresponds to the asymmetric stretching vibration of the same bond, providing evidence of the presence of NiO in the sample. The small peak at 1029 cm^−1^ corresponds bending vibration of the Ag–O bond, while the intense peak at 1056 cm^−1^ corresponds to the stretching vibration of the same bond, indicating the presence of Ag in the sample. Finally, the peak at 1097 cm^−1^ corresponds to the asymmetric stretching vibration of the Cu–O bond, which is a characteristic peak of Cu–O. The peaks detected in the Raman spectra can be used to determine the crystallinity and composition of the nanoparticles.

### Electrochemical Evaluation of HER electrocatalysts in acidic media

**Figure 5:**
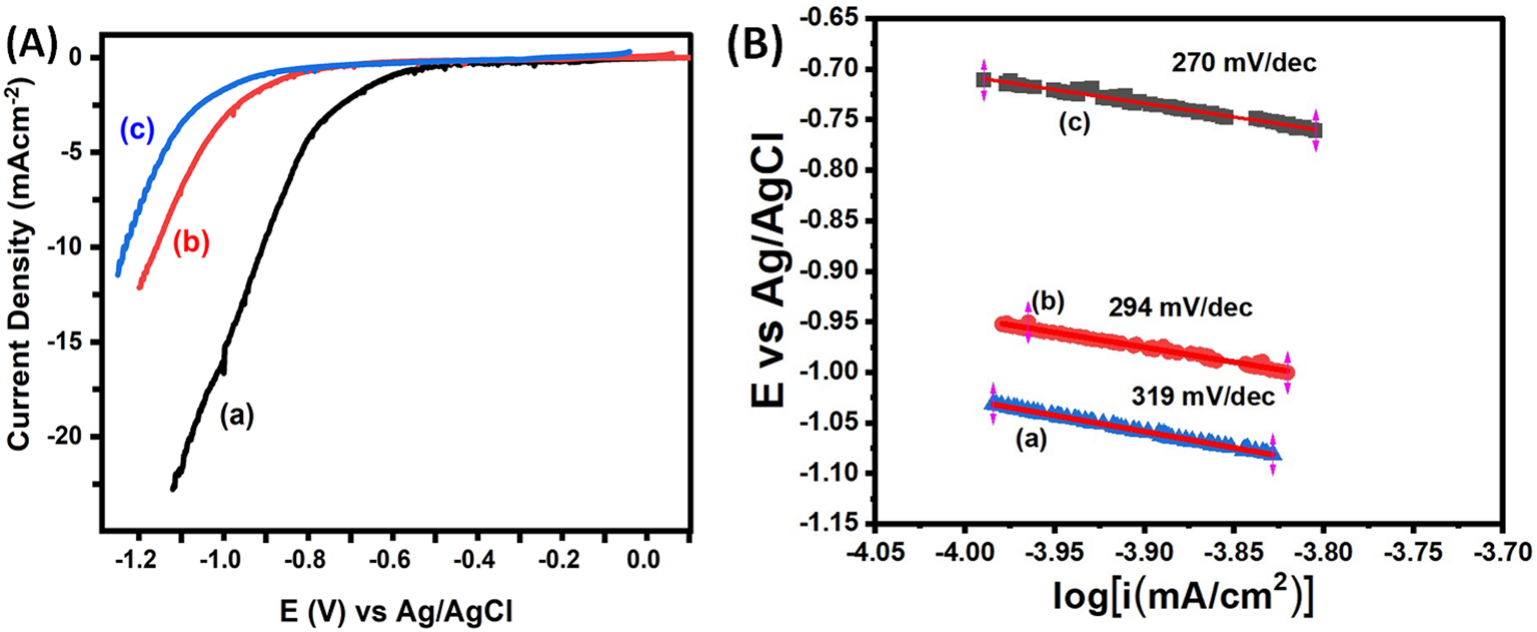
Electrocatalytic HER experiment in 0.5 M H_2_SO_4_ with Ag/AgCl (WE) and Pt coil as RE and CE (**A**) LSV comparison of all the (a) TMSiNPs-GCE (b) BMSiNPs-GCE and (c) bare-GCE. (**B**) Tafel plots were constructed based on the polarization curves obtained in (a) bare-GCE (b) BMSiNPs and (c) TMSiNPs.

The LSV technique was used to examine the Electrochemical performance of composite materials based on BMSiNPs and TMSiNPs for HER. The LSV was carried out at 5 mV s^−1^ scan rate with 0.5 M H_2_SO_4_ electrolyte. The polarization curves for cathodic reactions of all samples are presented in Fig. 5A.

**Figure 6:**
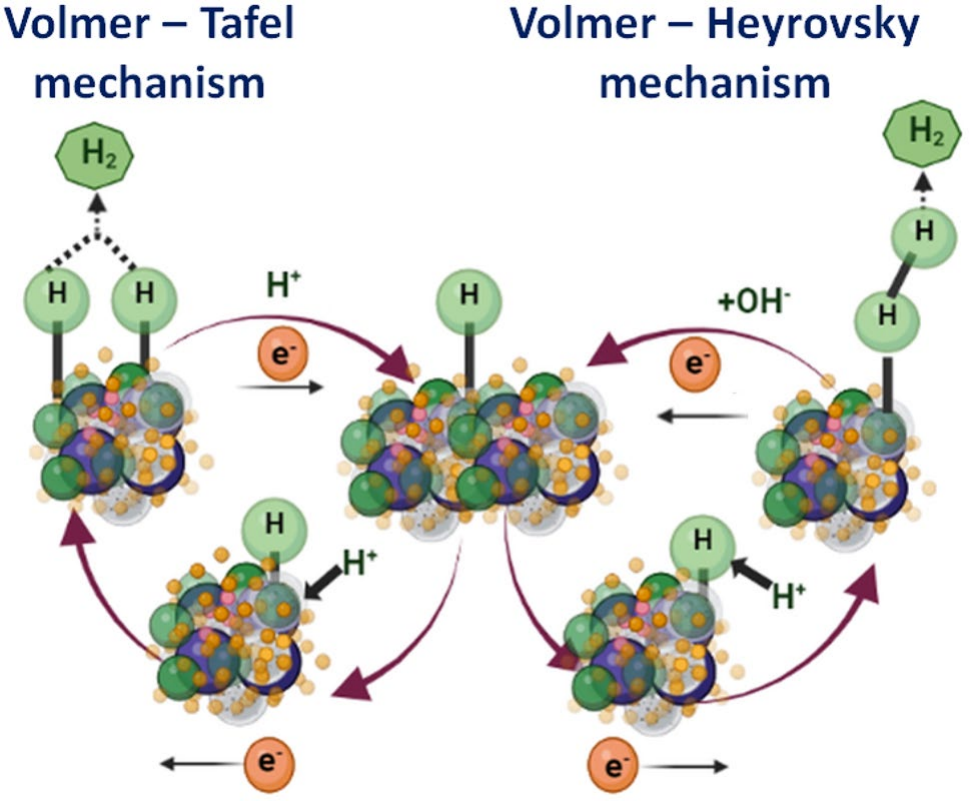
H_2_ evolution pathway mechanism in acidic medium.

The electrochemical production of hydrogen involves multiple steps that occur on the surface of the catalyst (Fig. 6). In acidic conditions, the reaction is believed to follow these steps [Eqs. (2)–(4)],

2 $$(\mathrm{Volmer \, step}) \quad \quad {H}^{+}+M+ {e}^{-}\leftrightarrow M- {H}^{*}$$

3 $$(\mathrm{Heyrovsky \, step}) \quad \quad M-{H}^{*}+{H}^{*}+ {e}^{-}\leftrightarrow M- {H}_{2}$$

4 $$(\mathrm{Tafel \, step}) \quad \quad 2\left[M-{H}^{*}\right]\leftrightarrow 2M+ {H}_{2}$$

Here, H* denotes a chemisorbed hydrogen atom located on the active site of the electrocatalyst (M). While the first step is common, the evolution of hydrogen can proceed through either of the latter two steps. To determine the most likely pathway for H_2_ generation, the Tafel slope is commonly utilized. The theoretically calculated Tafel slope for Pt, a model catalyst, is 29 mV dec^−1^ assuming that the Tafel step is the rate-determining step (RDS) according to the Butler-Volmer equation. If the Heyrovsky step is the RDS, then the Tafel slope should be 38 mV dec^−1^. However, if the first step, i.e., discharge or the Volmer step, is the slowest, then the Tafel slope should be 116 mV dec^−1^ regardless of whether H_2_ evolution occurs via the latter two steps. It is important to highlight that a smaller Tafel slope value is indicative of a more efficient catalyst for the HER.

To facilitate comparison, the dataset includes information for 40% Pt/C and the bare-GCE. It was observed that the GCE had significantly lower activity compared to TMSiNPs-GCE and Pt/C polarization curves. The onset potential for HER was determined to be − 0.9 V for BMSiNPs-GCE and − 0.7 V (vs Ag/AgCl) for TMSiNPs-GCE, whereas for Pt/C it was − 0.23 V (Ag/AgCl)^26^.

The Tafel slope results obtained for the bare-GCE, BMSiNPs-GCE, and TMSiNPs-GCE Fig. 5B indicate that the electrochemical reaction kinetics are influenced by the presence of the composite materials. The lower tafel slope value obtained for the TMSiNPs-GCE is 270 mV dec^−1^ compared to the BMSiNPs-GCE is 294 mV dec^−1^ can be associated to the presence of NiO, which enhances the catalytic activity. The maximum current density achieved indicates that the composite materials have good stability and can be used for efficient hydrogen production.

**Figure 7:**
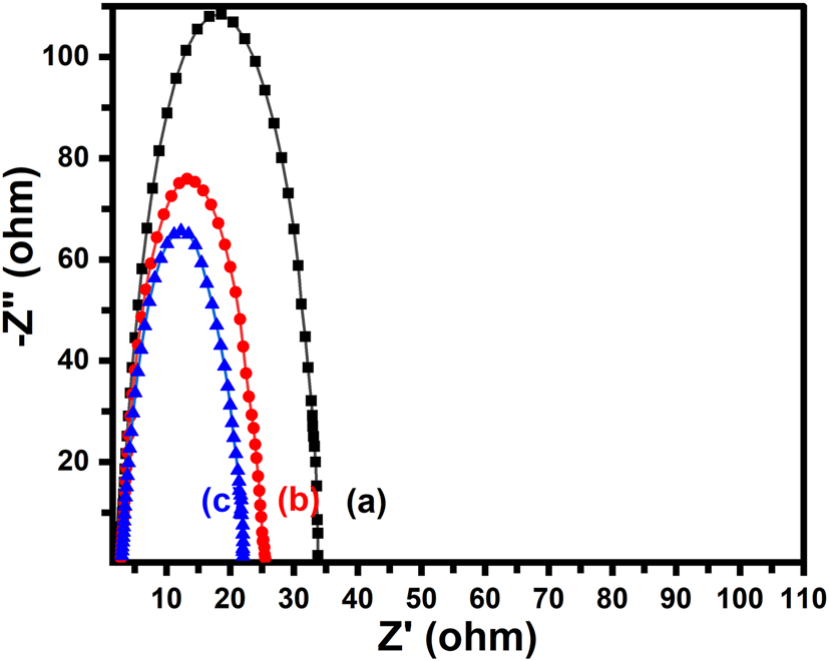
Nyquist plot of (a) bare-GCE, (b) BMSiNPs-GCE and (c) TMSiNPs-GCE at the onset potential.

To explore the characteristics of the TMSiNPs interface and improve the electrical interaction amongst the electrode and solution, electrochemical impedance spectroscopy (EIS) was conducted at the initial potential of the sample, covering a frequency range from 10^5^ to 1 Hz, using an alternating current voltage of 5 mV. The Nyquist plots of the samples are presented in Fig. 7. The TMSiNPs exhibited a charge transfer resistance (R_ct_) of 12.0 Ω in comparison to BMSiNPs-GCE exhibited (R_ct_) of 32 Ω, indicating a faster rate of electron transfer, which resulted in the acceleration of HER kinetics. This observation was further supported by the Tafel slope values and polarization curves.

The electrocatalytic efficacy of BMSiNPs, TMSiNPs in HER was studied using cyclic voltammetry (CV) at various scan rates (10 mVs^−1^ to 150 mVs^−1^) in the non-faradic region to assess the influence of electrochemical double layer capacitance (C_dl_). Therefore, the electrochemical surface area (ECSA) of the catalyst's (Fig. S3A–D) determined by the double layer capacitance (C_dl_) is 10.9 and 19.2 (µF cm^−2^) for BMSiNPs and TMSiNPs respectively. The EIS and ECSA data clearly demonstrate that the trimetallic composite exhibits superior charge transfer and a higher ECSA compared to its bimetallic counterpart. These findings strongly support the notion that the enhanced catalytic activity of the trimetallic composite can be attributed to these factors. Table 1 presents a comparison of reported catalysts with Tafel slopes for HER in acidic medium^27–30^.

**Table 1:** A table of similarity assessments of the reported catalysts with Tafel slope for HER.

S. no.	Electrocatalyst	Reaction condition	Tafel slope (mV dec^−1^)	References
1	TMSiNPs	H_2_SO_4_	270	This work
2	BMSiNPs	H_2_SO_4_	294	This work
3	CuS-Au and CuS	H_2_SO_4_	179 and 449	^27^
4	CuS/CoS_2_	H_2_SO_4_, KOH and PBS	142 and 183 mV	^28^
5	Ni(OH)_2_@CuS	KOH	186, 204	^29^
6	Ag_2_S/CuS	H_2_SO_4_	193	^30^

### Photocatalytic hydrogen evolution

**Figure 8:**
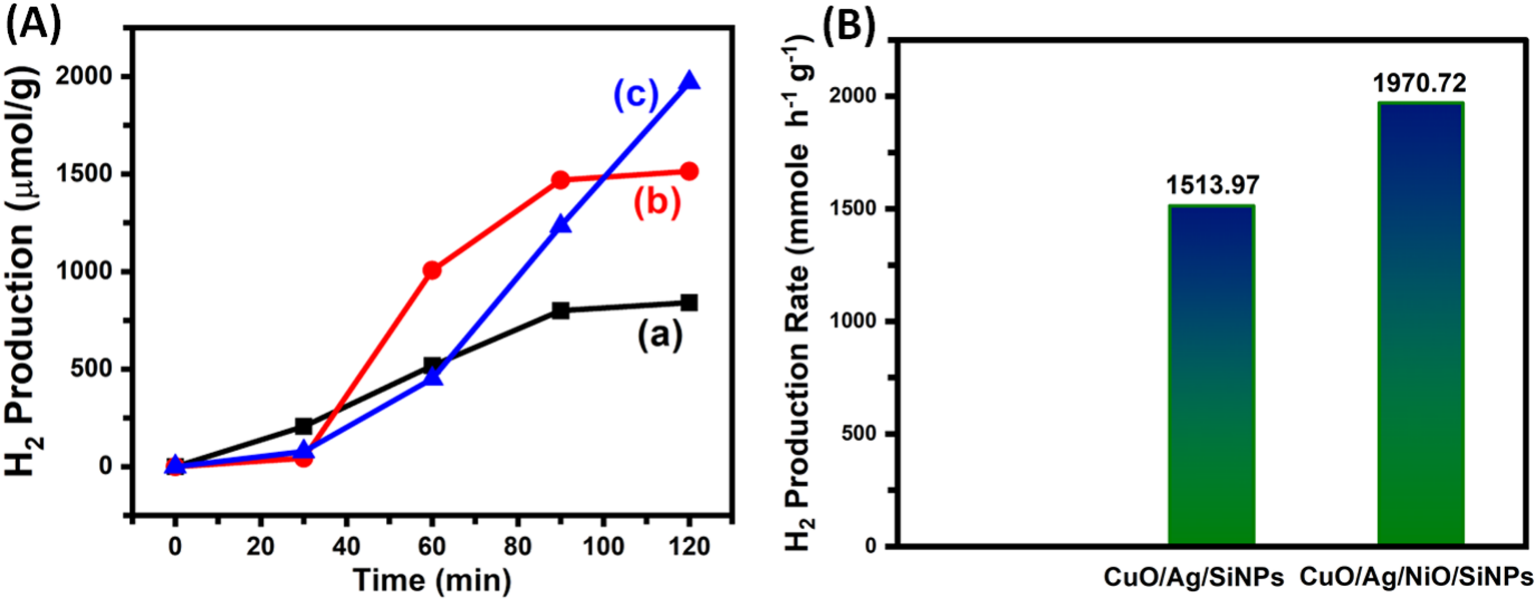
Photochemical H_2_ evolution activity of the catalysts: (**A**) time-yield curves and (**B**) H_2_ production rate.

**Figure 9:**
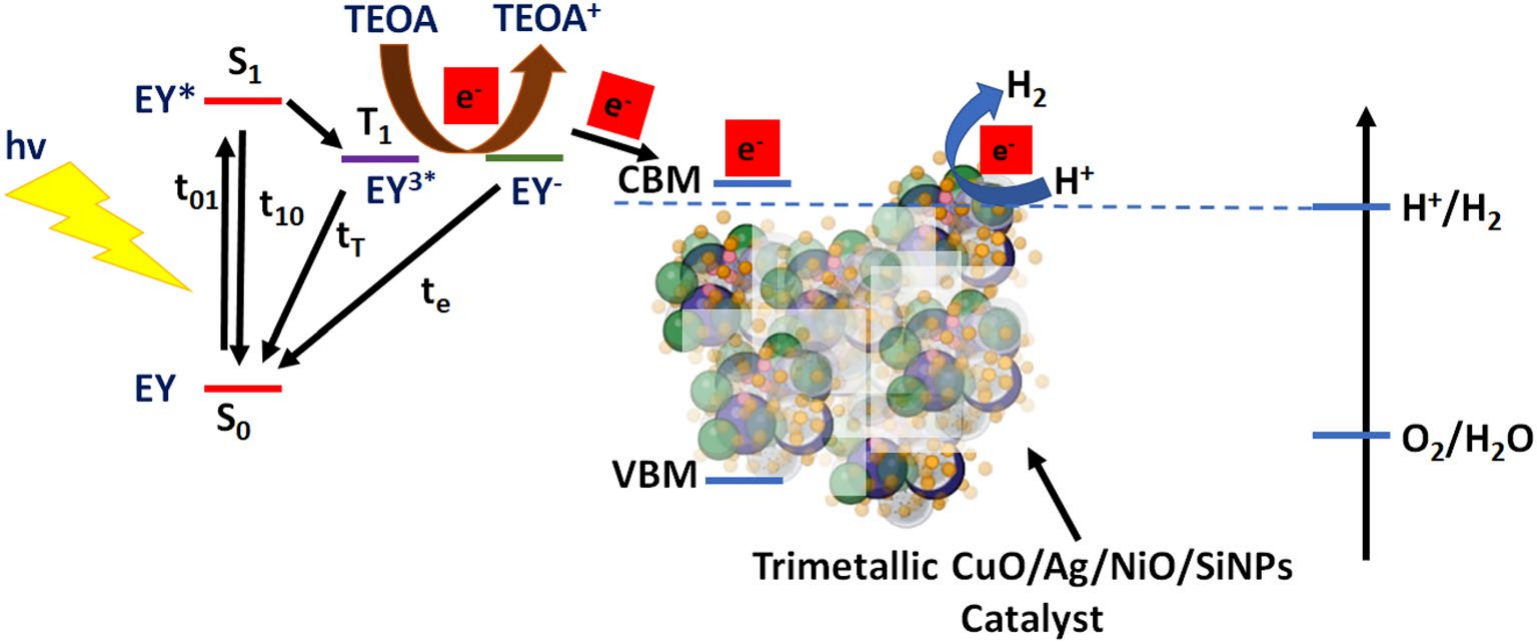
Evolution reactivity schematic representation using TMSiNPs photocatalyst.

The synthesized BMSiNPs and TMSiNPs composites were investigated for their potential in dye-sensitized photochemical water splitting. The water splitting activities of the bimetallic and trimetallic composites were determined to be 1513.97 and 1970.72 (mmol h^−1^ g^−1^) respectively (Fig. 8A,B), showcasing their promising capabilities in this application. Figure 9^31^ illustrates the mechanism used for dye-sensitized HER. The process initiates with the absorption of light via Eosin Y (EY), resulting in the production of a photoexcited dye molecule (EY*). This photoexcited state then undergoes intersystem crossing to generate a long-lived excited state known as EY^3^*. Subsequently, EY^3^* accepts an electron from TEOA through a process called reductive quenching, leading to the formation of EY^−^. The electron originating from EY^−^ is subsequently transferred to the catalyst, where the process of water reduction takes place. Several aspects play a crucial role in the water reduction process on the catalyst's surface, including efficient light absorption by the dye molecule, effective separation of charge carriers, and efficient electron transfer to the reduction site. In our specific system, the dye molecule exhibits high light absorption capabilities, allowing it to effectively harness solar radiation and transfer the electron to the TMSiNPs catalyst layers. The photochemical hydrogen evolution reaction (HER) performance of TMSiNPs compares to that of the BMSiNPs material substantially. TMSiNPs, in specifically, exhibits an apparent quantum yield (AQY) of 21.88%, suggesting its excellent efficiency in this procedure.

After the electron is transferred to the composite materials, the potential difference between the metal and semiconductor nanoparticles causes charge separation, where the excited electrons are relocated to the metal nanoparticles and the holes are transferred to the oxygen molecule in the surrounding environment, creating hydroxyl radicals. The hydroxyl radicals react with water molecules (H_2_O) to form hydrogen gas (H_2_) and oxygen molecules (O_2_). The metal and semiconductor nanoparticles regenerate and continue the photocatalytic process, providing a sustainable source of hydrogen gas. The EIS studies discussed in the previous section explains that the trimetallic composite exhibits better charge transfer properties than the bimetallic counterpart and therefore exhibits better hydrogen evolution rates. Table 2^32–34^, presents a comprehensive comparison of various Cu-related ternary photocatalysts, focusing on their performance in the photocatalytic production of hydrogen.

**Table 2:** Data comparing various photocatalysts for hydrogen (H_2_) evolution are presented.

Photocatalysts	Light source	Sacrificial reagents	Optimized HER (mmol h^−1^ g^−1^)	References
Cu@Cu_2_O/ZnO	Xe lamp	Na_2_S/Na_2_SO_3_	1472.2	^32^
Pt/CuO/TiO_2_	Xe lamp	Methanol	181.8	^33^
Cu/Cu_2_O/TiO_2_	Xe lamp	Methanol	45.56	^34^
BMSiNPs	Xe lamp	TEOA	1513.97	This work
TMSiNPs	Xe lamp	TEOA	1970.72	This work

## Conclusion

The globally carbon dioxide emissions, which are mostly driven by the production and use of grey and blue hydrogen, indicate a grave threat to our planet's climate. In response to this essential challenge, our study ventures into the domain of sophisticated bimetallic and trimetallic nanoporous materials, revealing their enormous potential for green hydrogen generation via water splitting. Our rigorous synthesis and characterization of BMSiNPs and TMSiNPs composite materials confirms their success, paving the way for a game-changing breakthrough. These catalysts, discovered via our research, have demonstrated nothing short of extraordinary performance, excelling in both electrocatalysis and photocatalysis. Our BMSiNPsGCE and TMSiNPsGCE catalysts, in particular, demonstrated outstanding onset potentials for the Hydrogen Evolution Reaction (HER) at − 0.9 V and − 0.7 V (vs Ag/AgCl), respectively.

Furthermore, the η@10 values of − 1.26 V for BMSiNPs − GCE and − 1.00 V for TMSiNPs-GCE, as well as the exceptionally low Tafel slopes of 294 mV dec^−1^ for BMSiNPs-GCE and 270 mV dec^−1^ for TMSiNPs-GCE, highlight these materials' outstanding catalytic abilities.

Notably, the TMSiNPs composite is blazing a path with a photochemical H_2_ evolution rate of 1970.72 mmol h^−1^ g^−1^, while the BMSiNPs composite shines brightly with a rate of 1513.97 mmol h^−1^ g^−1^.

Our thorough research, which included EIS and ECSA measurements, backs up these ground-breaking findings, indicating lower resistance to charge transfer and an enlarged ECSA in the trimetallic catalyst. Apart from its scientific value, this finding provides a cost-effective path toward the creation of HER electrocatalysts that are both sustainable and efficient.

### Supplementary Information

Supplementary Information.

## Data Availability

All data generated or analysed during this study are included in this published article and its supplementary information files.
