# Supplementary material for: Trimetallic CuO/Ag/NiO supported with silica nanoparticles based composite materials for green hydrogen production
Source: Sci Rep. 2023 Oct 7;13:16909. doi: 10.1038/s41598-023-43697-4 (PMC10560267; doi:10.1038/s41598-023-43697-4)
Supplement: Supplementary file 1 — Supplementary Information. [file 41598_2023_43697_MOESM1_ESM.docx]

# Trimetallic CuO/Ag/NiO Supported with Silica Nanoparticles Based Composite Materials for Green Hydrogen Production

# Gowhar A. Naikoo^1^*, Mustri Bano^1^*, Israr U. Hassan^2, +^, Mohd Monis Ayyub^3^*^, +^ and Mona Zamani Pedram^4*^

^1^Department of Mathematics & Sciences, College of Arts & Applied Sciences, Dhofar University, PC 211, Salalah, Oman

^2^College of Engineering, Dhofar University, PC 211, Salalah, Oman

3New Chemistry Unit and School of Advanced Materials, Jawaharlal Nehru Centre for Advanced Scientific Research, Bangalore 560064, India

^4^Faculty of Mechanical Engineering-Energy Division, K.N. Toosi University of Technology, P.O. Box: 19395-1999, No. 15-19, Pardis St., Mollasadra Ave., Vanak Sq., Tehran 1999 143344, Iran

*[*gahmed@du.edu.om*](mailto:gahmed@du.edu.om)*,* [*mustribano1@gmail.com*](mailto:mustribano1@gmail.com)*,* *mmonis16@gmail.com,m.zpedram@kntu.ac.ir*

+these authors contributed equally to this work

**Trimetallic CuO/Ag/NiO Supported with Silica Nanoparticles Based Composite Materials for Green Hydrogen Production**


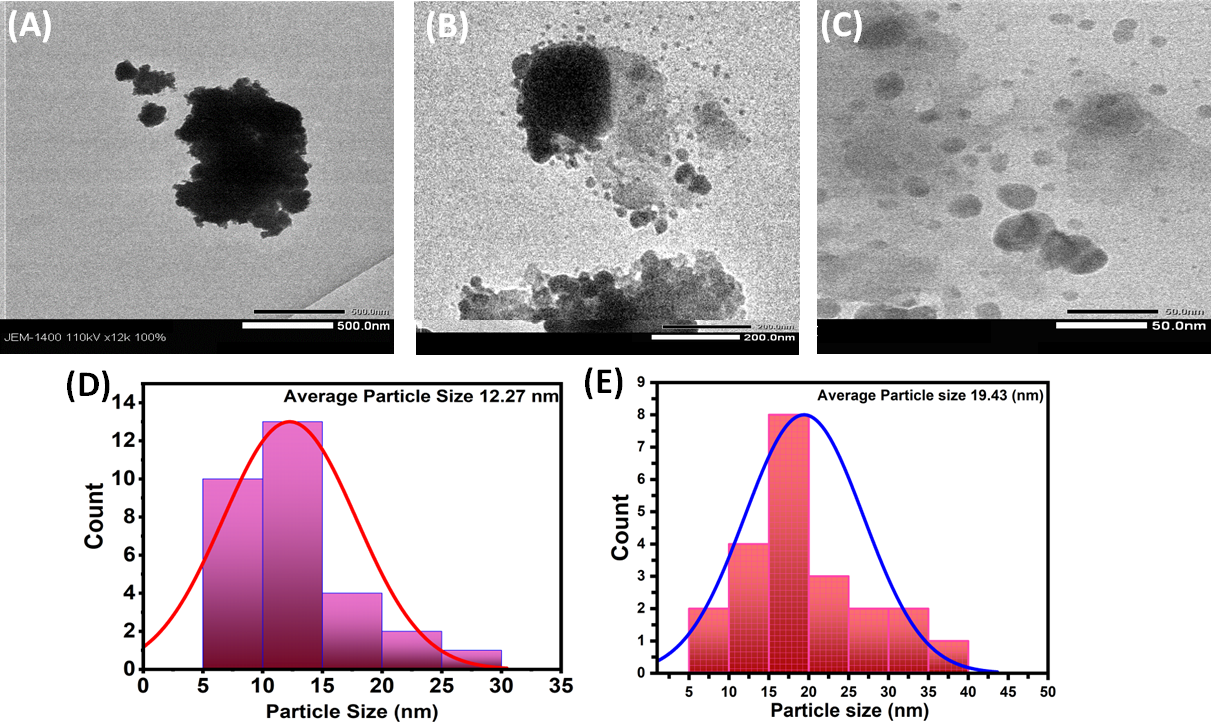


**Fig. S1 TEM images of (A) CuO/Ag/SiNPs at 500 nm, (B) & (C) CuO/Ag/NiO/SiNPs at 100 µm and 50 nm. (D) and (E) size histograms of CuO/Ag/NiO/SiNPs image (B) and (C) respectively.**


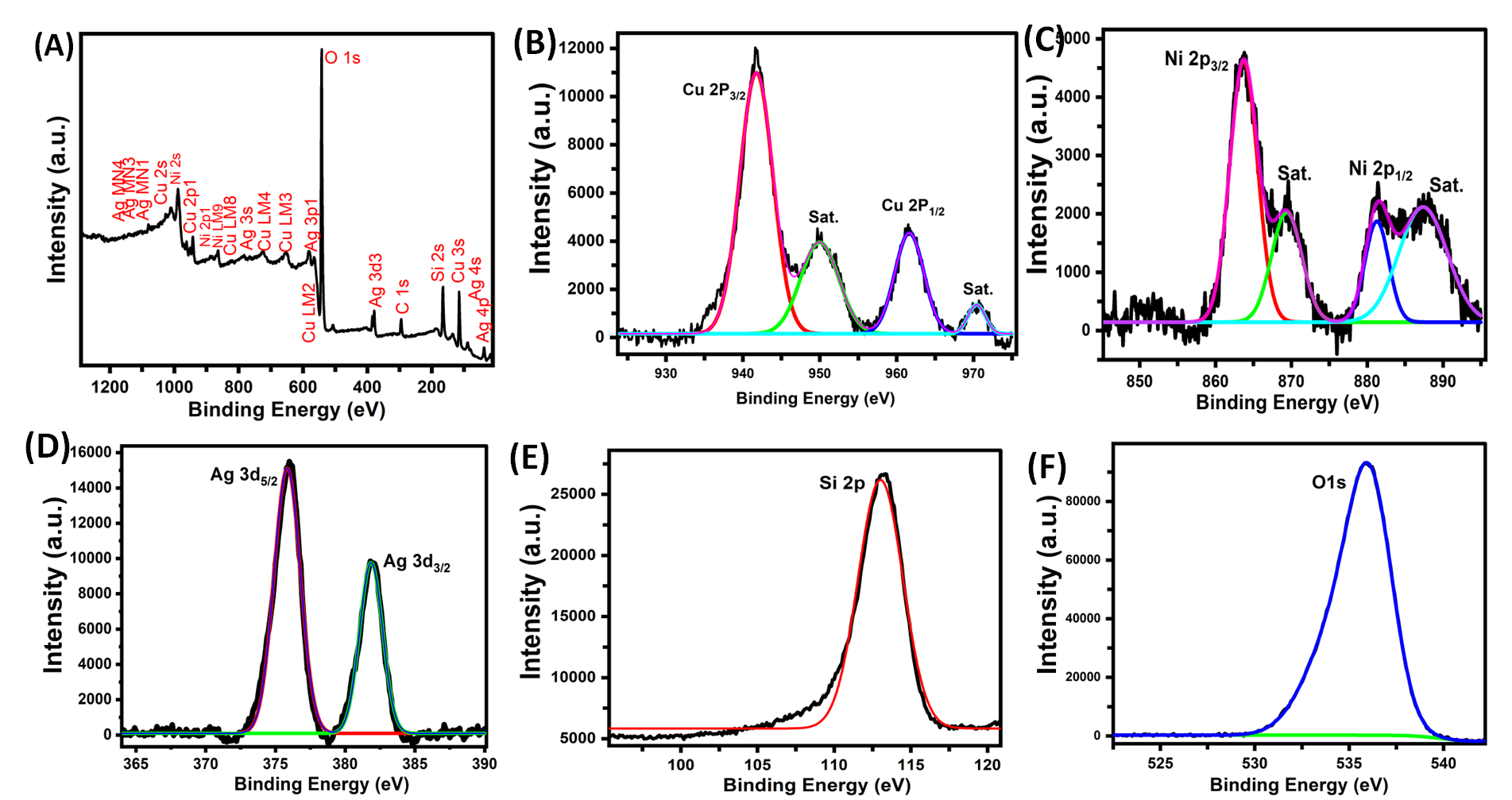


**Fig. S2 XPS spectra of CuO/Ag/NiO/SiNPs (A) wide spectrum, (B) Cu 2p spectrum (C) Ni 2p spectrum (D) Ag 3d spectrum (E) Si-O 2p spectrum (F) O 1s spectrum.**

The graph would show the binding energy on the x-axis and the signal intensity on the y-axis. Each element would have its characteristic peaks at specific binding energies, which can be used to identify the elements present in the nanoparticles. The relative intensities of the peaks would provide information about the elemental composition of the nanoparticles. Additionally, the XPS spectra can be analysed to determine the oxidation state of the elements present in the nanoparticles.


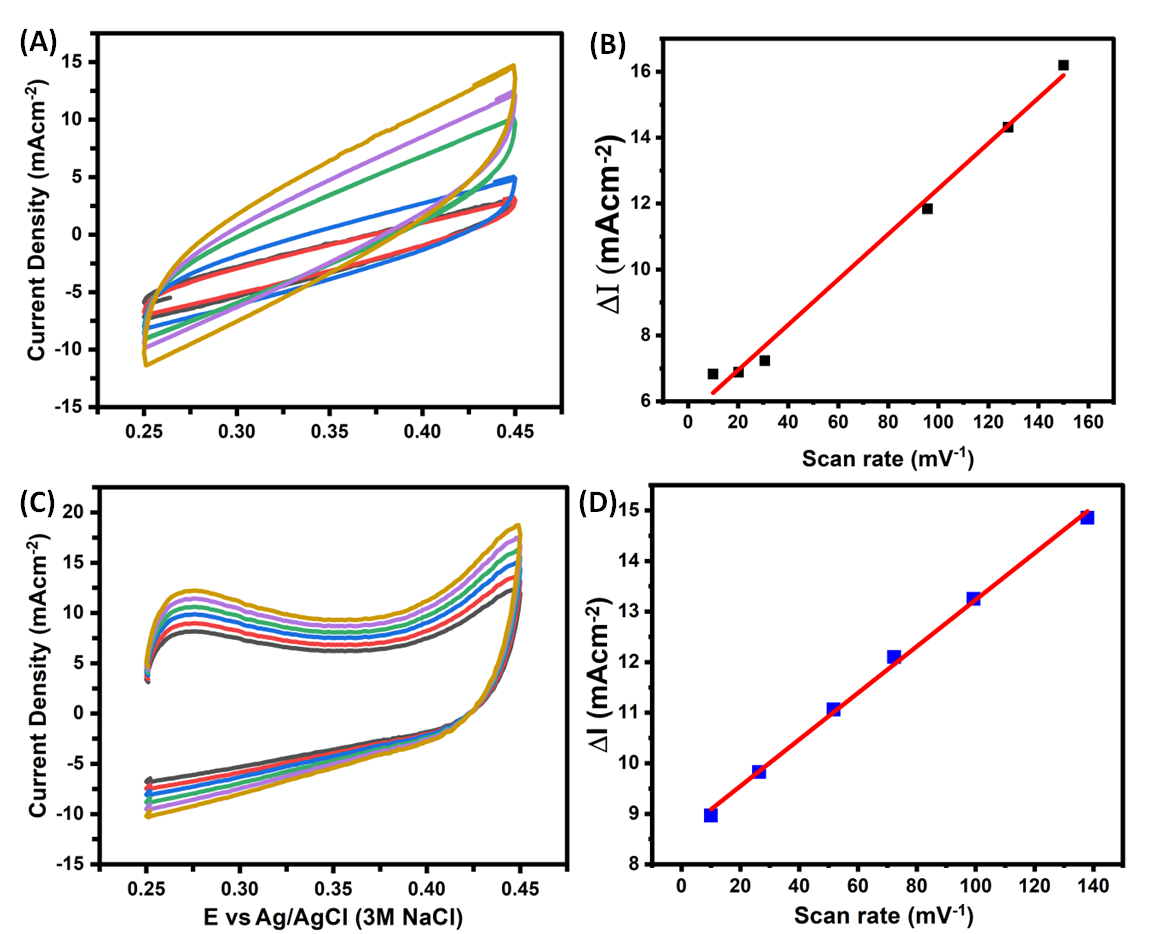


**Fig. S3 CV Curves of (A)** CuO/Ag/SiNPs-GCE **and (C)** CuO/Ag/NiO/SiNPs-GCE are the corresponding difference in the current density at 0.25 V plotted against scan rate **(B) and (D)** to calculate the Cdl value form the slope after the linear fit.

**Calculation of double layer capacitance (C_dl_):**

In studies on electrochemical catalysts for HER, their activities are often analyzed through normalization by either mass or electrochemically active surface area. To determine the capacitance of the electrode, we utilized a CV method and found that its value is directly proportional to the electrochemically active surface area. Capacitance measurements were conducted within a potential range of 0.25-0.45 V to ensure that no faradaic process related to HER took place and that the electrode acted as an ideally polarizable electrode (IPE). The electrode's current changes over time in response to a certain voltage ramp (V/s).

$$i=vc_{d}[1-\exp(-\frac{t}{R_{s}C_{d}})]$$

where $R_{s}$is solution resistance. $vC_{d}$ is saturation current at large time. Thus, plot of Δ𝐼 |𝑖𝑎 - 𝑖𝑐 | at 0.35 V against scan rate gives a straight line with slope twice the value of C_dl_.

**Tafel slope:**

The electrochemical production of hydrogen is typically a complex process that takes place on the surface of a catalyst.

One critical factor in determining a catalyst's inherent activity is the exchange current density (I_0_), which refers to the current density in one direction (either cathodic or anodic) at the equilibrium potential of a reaction. This parameter describes the rate of electron transfer under reversible conditions and is related to the overpotential. By applying the Tafel equation, it is possible to calculate both the io and the charge transfer coefficient (α).

$$\eta=a -blog (\frac{i}{i_{0}})$$

**Where** $'b^{'}=-\frac{2.303RT}{\alpha F}$ and is the Tafel slop, $'a^{'}=-\frac{2.303RT}{\alpha F} log(i_{0})$.

**Apparent quantum yield calculation:**

The apparent quantum yield is calculated by taking into account the total number of incoming photons applied in the photochemical process (the complete spectrum in which the photocatalyst is active). The photon flux (number of photons entering the reaction cell) was estimated using a New Port irradiance meter.

The number of incident photons were calculated integrating photons available from 395 nm (λ >395 nm, UV cut-off filter used to allow only visible-light photons) to 512 nm (absorption edge or cut-off wavelength).[1] From the lamp spectra number of photons

Number of photons entering cylindrical sample cell (diameter = 4.5 cm) = $\int_{395}^{517} Fd\lambda=7.53 \times{10}^{18} photons /second$

HER activity of BMSiNPs = 1513.97 mmol/h/g = 0.2532 $\times$ 10^18^ H2/second

$$AQY \left( \% \right)=2 \times\frac{Number of evolved hydrogen}{Number of incident photons} \times100=2 \times\frac{0.2532 \times{10}^{18}}{7.53\times{10}^{18}} \times100=67.27 \%$$

HER activity of TMSiNPs = 1970.72 mmol/h/g = 0.3296 $\times$ 10^18^ H2/second

$$AQY \left( \% \right)=2 \times\frac{Number of evolved hydrogen}{Number of incident photons} \times100=2 \times\frac{0.3296 \times{10}^{18}}{7.53\times{10}^{18}} \times100=87.57 \%$$

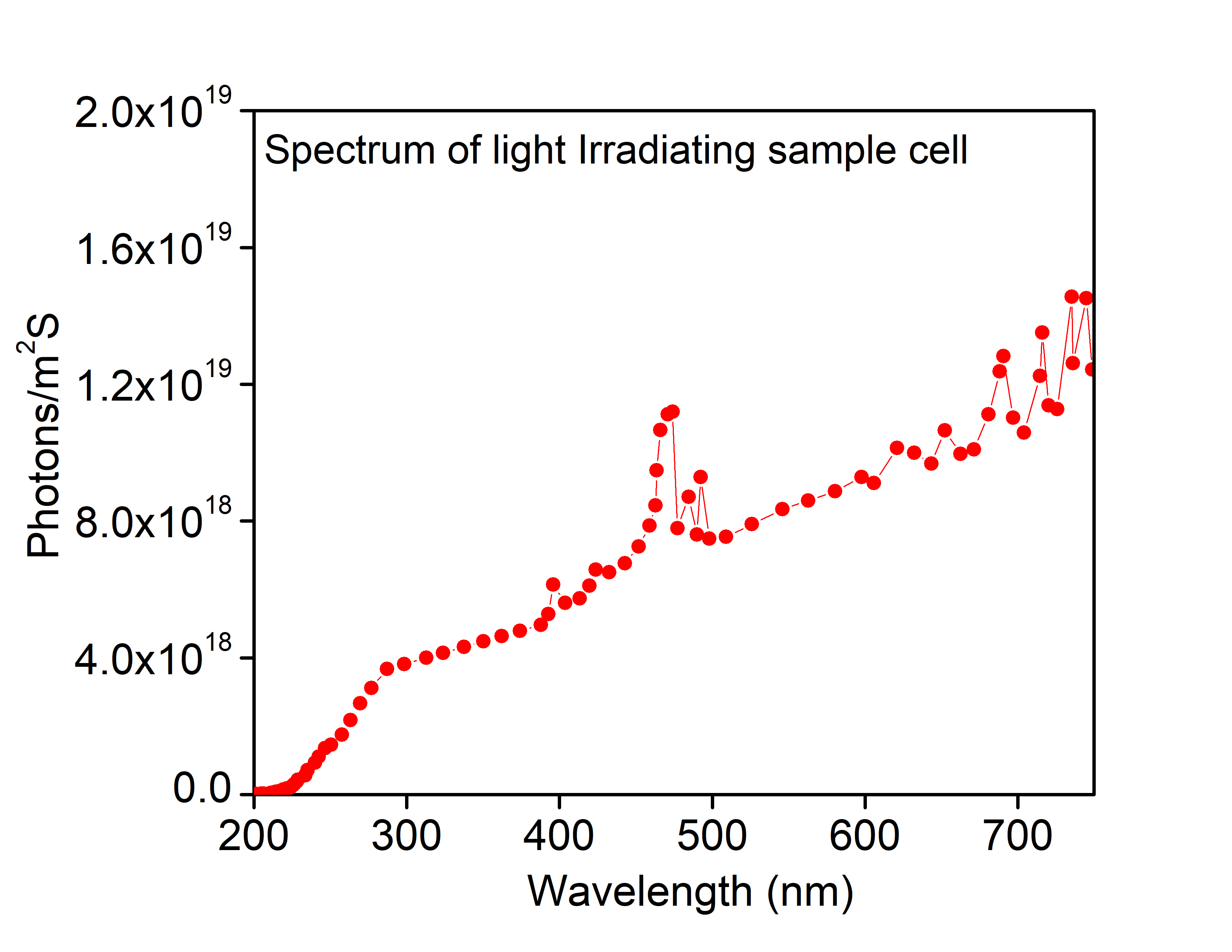


**Fig. S4.** **The light-source spectrum employed in the HER process.**

**References**

1. A. Roy, A. Singh, S. A. Aravindh, S. Servottam, U. V. Waghmare and C. N. R. Rao, Angew. Chem. Int. Ed., 2019, 58, 6926-6931.
